# Panhypopituitarism Secondary to a Central Nervous System Germinoma

**DOI:** 10.7759/cureus.98887

**Published:** 2025-12-10

**Authors:** Somia Hassanes, Eboh Cecil, Betsy Francis

**Affiliations:** 1 Medicine, University Hospitals Sussex NHS Foundation Trust, Chichester, GBR

**Keywords:** central diabetes insipidus (cdi), germinoma, hypopituitarism (hp), intracranial germ cell tumors, pineal germinoma, suprasellar tumour

## Abstract

Germ cells are the reproductive cells in a foetus which later develop into sperm in the testicles or unfertilized eggs in the ovaries. They occur in the gonads (testis and ovaries) but can also occur elsewhere in the body, including intracranially. In this case, these tumours can affect the function of the pituitary gland, causing different endocrinological conditions. Understanding the aetiology, classification, and clinical behaviour of germ cell tumours is essential for timely diagnosis, appropriate treatment, and prognostication, given the spectrum of entities encompassed by this group and their variable therapeutic sensitivity and outcomes. This case report highlights the importance of considering intracranial germ cell tumours in a young adult admitted with endocrinopathy as the first presentation.

## Introduction

Germ cell tumours (GCTs) are neoplasms arising from primordial germ cells that fail to complete their normal migration or differentiation during embryogenesis [1]. While they most commonly develop in the gonads (testes and ovaries), extragonadal GCTs can occur along midline structures, including the mediastinum, retroperitoneum, and central nervous system (CNS) [1,2]. The pathogenesis of extragonadal GCTs is thought to involve ectopic germ cells that persist along their migratory pathway, ultimately giving rise to neoplasms in aberrant locations [1].

Histologically, GCTs are broadly classified into seminomatous (germinomatous) and non-seminomatous (non-germinomatous) types [3]. Seminomatous tumours are characterised by a uniform population of undifferentiated germ cells, clear cytoplasm, and a prominent lymphocytic infiltrate, and are highly sensitive to radiotherapy and chemotherapy [3]. Non-germinomatous GCTs often secrete tumour markers such as alpha-fetoprotein (AFP) or beta-human chorionic gonadotropin (β-HCG) and typically require multimodal therapy due to relative resistance to single-modality treatment [4].

GCTs display distinct epidemiological patterns. Testicular GCTs are the most common malignancy in young adult males, whereas ovarian GCTs are rare and usually present in children or adolescents [1,2]. Extragonadal GCTs account for fewer than 10% of all GCTs, with the mediastinum being the most frequent site, followed by retroperitoneal and CNS locations [1,5]. Clinical presentation varies with tumour site and histology, ranging from painless masses or abdominal distension to systemic manifestations, including hormonal dysfunction or paraneoplastic syndromes [1,3].

Accurate diagnosis relies on imaging, histopathology, and tumour marker assessment [3,4]. Tumour markers, particularly AFP and β-HCG, are critical for diagnosis, risk stratification, and monitoring of treatment response, especially in non-seminomatous tumours [3]. Management strategies are tailored according to tumour location, histology, and stage, and typically involve surgery, chemotherapy, and radiotherapy, alone or in combination [4].

## Case presentation

A 22-year-old male was brought to the emergency department with a one-week history of feeling generally tired, vomiting recurrent syncopal attacks. The trigger for which was simply standing up, he would then feel lightheaded, fall to the ground, and lose consciousness. This was against the background of a 12-month history of loss of appetite and an unintentional 15.8 kg weight loss. He had a history of depression and was on citalopram 20 mg. He was a non-smoker and drank only occasionally with no history of illicit drug use. He lived with his family and was fully independent. History from the mother revealed a normal delivery, normal developmental milestones, normal puberty, and achieved a good height of 6 feet. There was no family history of significance.

On examination, he was conscious, could recall the sequence of events clearly, dehydrated, lethargic, apyretic, with sallow skin but no hyperpigmentation, and some axillary and pubic hair. Hemodynamically, he had a blood pressure of 94/65 mmHg and a heart rate of 103 beats per minute. Intact visual fields to confrontation and a normal sense of smell were noted. Finally, his chest, cardiovascular, and abdominal examination were all unremarkable. Table 1 shows his initial venous blood gas results. Table 2 shows his initial blood test results.

**Table 1:** Venous blood gas results.

Parameter	Result	Normal range
pH	7.483	7.350–7.45
Bicarbonate	23 mmol/L	22–29 mEq/L
Blood glucose	4.9 mmol/L	3.9–5.8 mmol/L
Lactate	1.4 mmol/L	05–1.6 mmol/L
Base excess	0.2 mmol/L	− 2 to 2 mmol/L

**Table 2:** Blood results. HBA1c: glycated hemoglobin; CRP: C-reactive protein; TSH: thyroid-stimulating hormone; T4: thyroxine; FSH: follicle-stimulating hormone; LH: luteinizing hormone; ACTH: adrenocorticotrophic hormone; IGF-1: insulin-like growth factor 1

Parameter	Result	Normal range
HbA1c	5%	<6.5%
CRP	1 mg/L	0–5 mg/L
Neutrophils	3.7 mg/L	2–7 × 10^9^/L
White blood cells	8.8 × 10^9^/L	4–10 × 10^9^/L
Hemoglobin	119 g/L	130–180 g/L
Troponin	<3.3 ng/L	0–34 ng/L
TSH	8.04 mu/L	0.3–4.9 mu/L
T4	6.7 pmol/L	9–19 pmol/L
Thyroid peroxidase antibodies	28.63 IU/mL	0–5.9 IU/ml
FSH	0.4 IU/L	1.3–19.3 IU/L
LH	<0.1 IU/L	1.7–8.6 IU/L
Testosterone	<0.17 nmol/L	8.64–29.0 nmol/L
Estradiol	<88 pmol/L	41.4–159 pmol/L
Random cortisol	<28 nmol/L	150–600 nmol/L
ACTH	13 ng/L	<50 ng/L
Adrenal antibodies	Negative	
Prolactin	842 mu/L	109–557 mu/L
Macroprolactin	Negative	
IGF-1	9.5 nmol/L	17.9–48.5 nmol/L
Growth hormone	0.1 ng/mL	<5 ng/mL

The endocrinologist reviewed the patient and advised a short synacthen test (SST) and an MRI of the pituitary. The results of the SST showed poor adrenal response, as shown in Table 3.

**Table 3:** Short Synacthen test results.

Bloods	Result	Normal range
Basal cortisol	-	>300 nmol/L
30-minute cortisol	165 nmol/L	>367 nmol/L
60-minute cortisol	219 nmol/L	>419 nmol/L

The endocrinologist’s impression was that of hypopituitarism, hypogonadotropic hypogonadism, and primary hypothyroidism (given raised thyroid-stimulating hormone (TSH) and positive thyroid peroxidase antibodies). He started the patient on testosterone replacement with Testavan 23 mg once a day, hydrocortisone 10 mg in the morning, 5 mg in the afternoon, and 5 mg in the evening, and he was provided with emergency steroid injections. He was educated regarding the sick day rule. He was booked for an MRI of his pituitary. As the patient was unable to tolerate the MRI due to claustrophobia, a CT scan of the pituitary was arranged instead.

**Figure 1:**
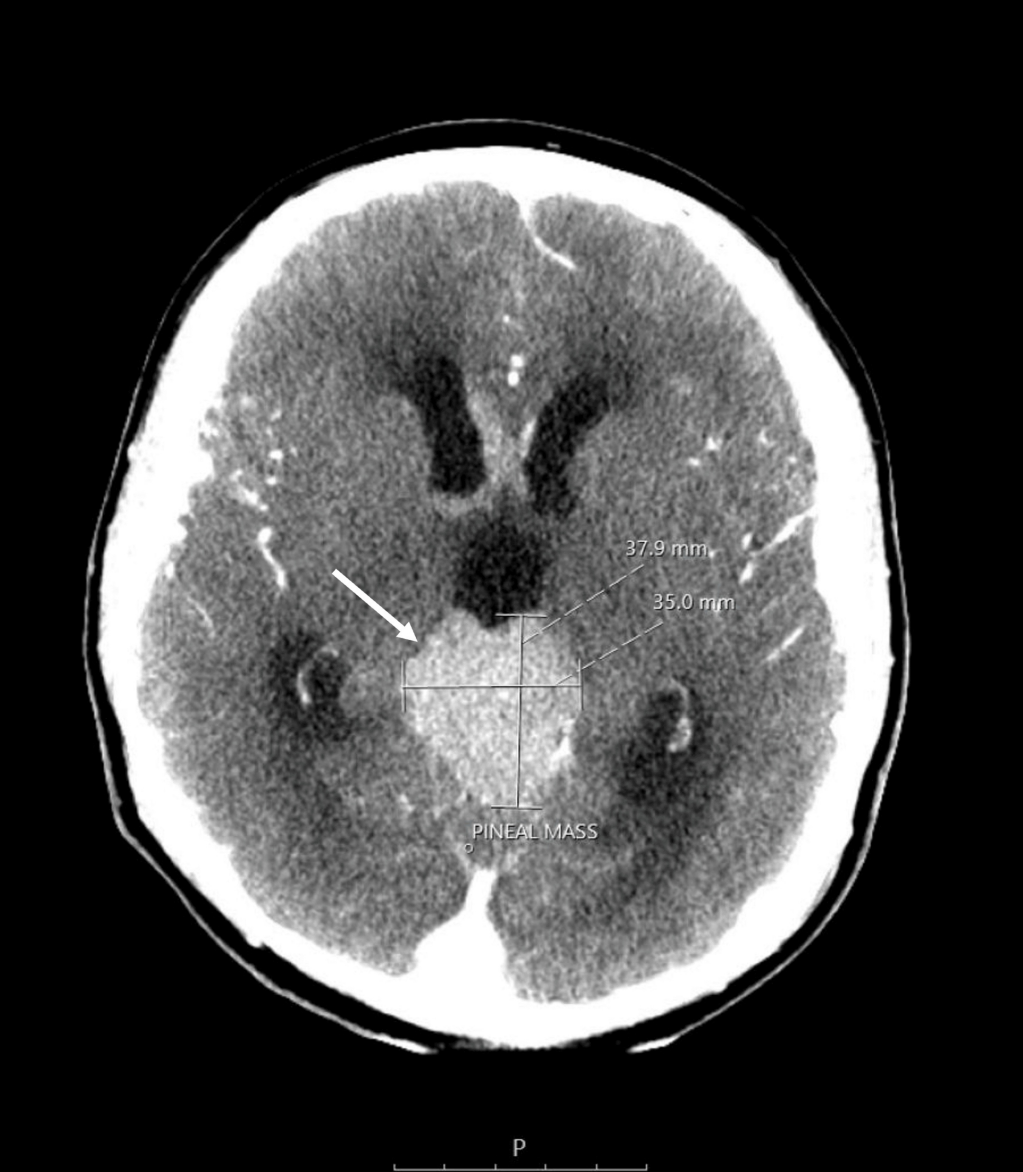
CT of the pituitary with contrast. White arrow: mass lesion in the pineal region.

**Figure 2:**
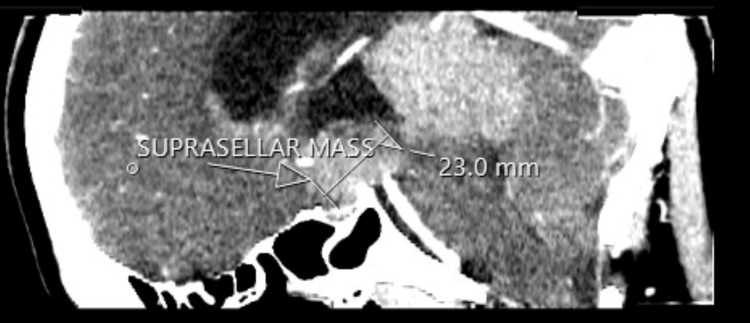
CT of the pituitary with contrast. White arrow: mass in the suprasellar region.

**Figure 3:**
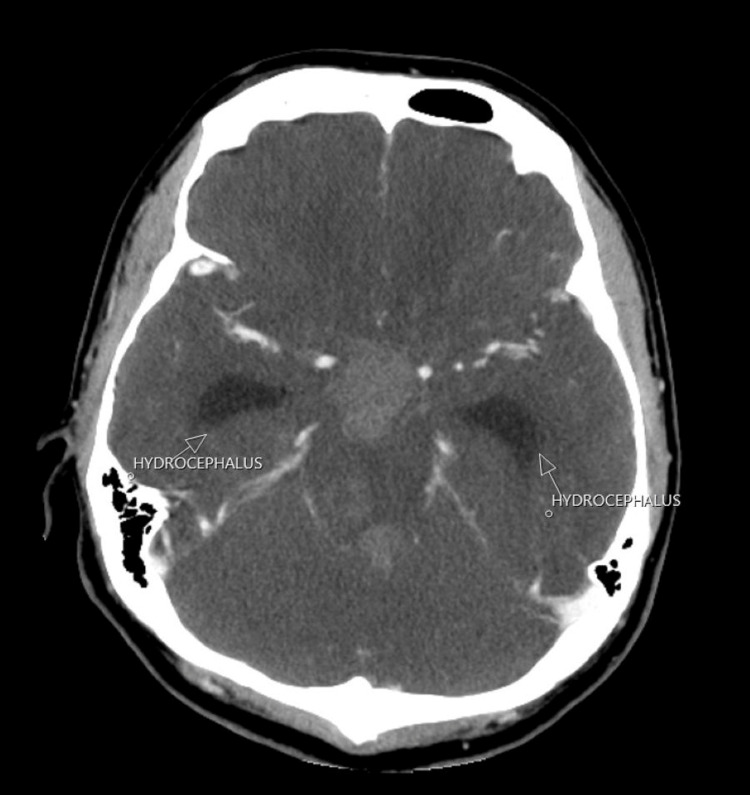
CT of the pituitary with contrast. White arrows: hydrocephalus.

**Figure 4:**
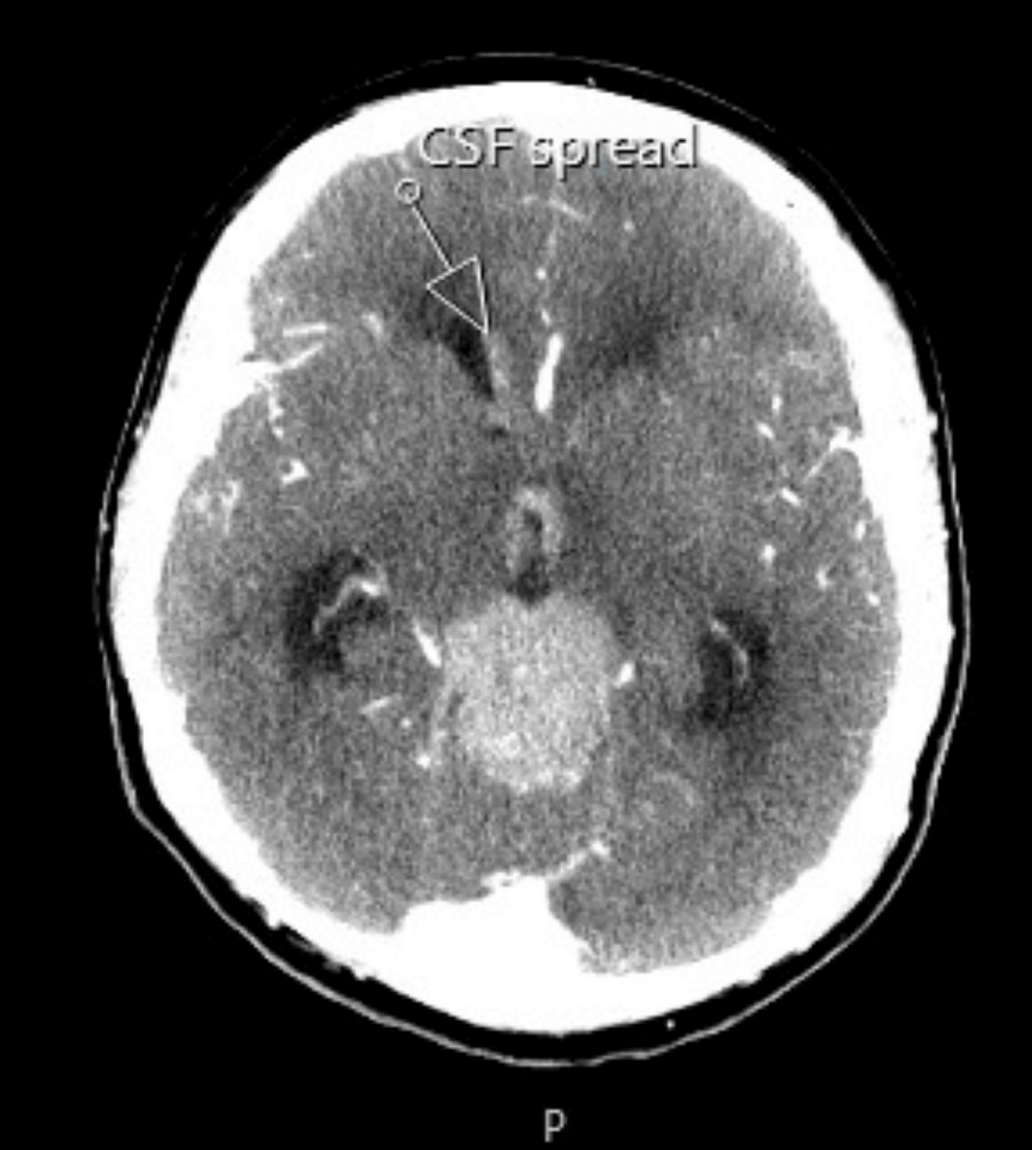
CT of the pituitary with contrast. White arrow: cerebrospinal fluid spread.

The CT scan of the pituitary showed a large avidly enhancing mass lesion in the pineal region, another similar synchronous enhancing mass lesion was seen in the suprasellar region, enhancing foci within the frontal horn of the right lateral ventricle extending along the midline septum and possibly within the fourth ventricle, and evidence of mass effect upon the midbrain with effacement of the cerebral aqueduct. Resultant supratentorial hydrocephalus with periventricular low-attenuation changes was consistent with cerebrospinal fluid (CSF) transudation (Figures 1-4).

The neurosurgical team was contacted with the results, and the patient was admitted under their care the same day. After starting the patient on hydrocortisone therapy, he complained of polyuria, urinating many times per day, and polydipsia, feeling thirsty all the time, and drinking about 6 L of water a day. He was started on desmopressin 50 µg three times a day, along with strict fluid input and output monitoring. His paired osmolalities are shown in Table 4.

**Table 4:** Paired serum and urine osmolality.

Test	Result	Normal range
Urine osmolarity	306 mOsm/kg	50–1,200 mOsm/kg
Serum osmolarity	322 mOsm/kg	275–295 mOsm/kg

**Figure 5:**
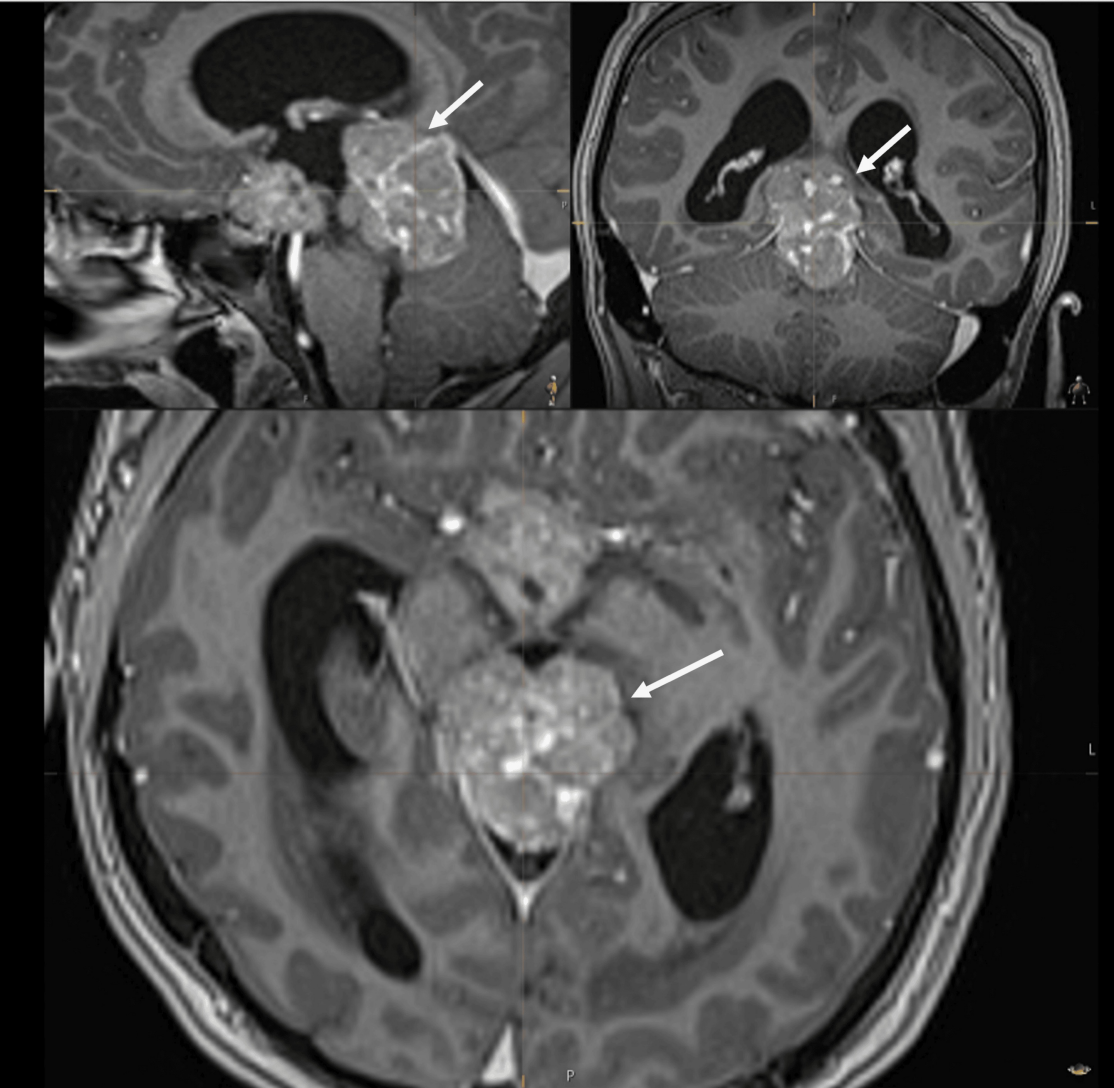
MRI of the head with contrast. White arrows: mass lesion in the pineal region.

**Figure 6:**
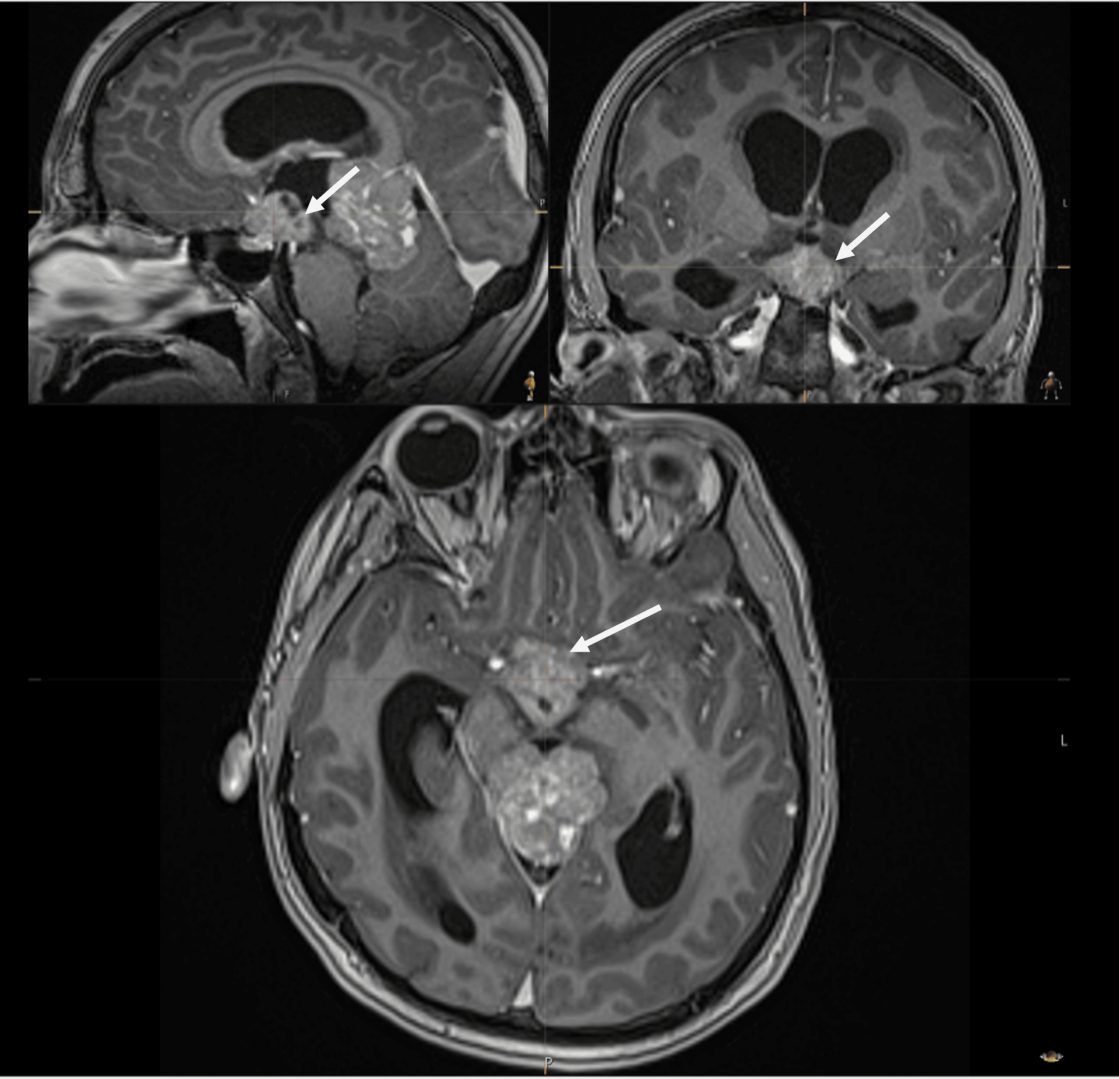
MRI of the head with contrast. White arrows: mass in the suprasellar region.

**Figure 7:**
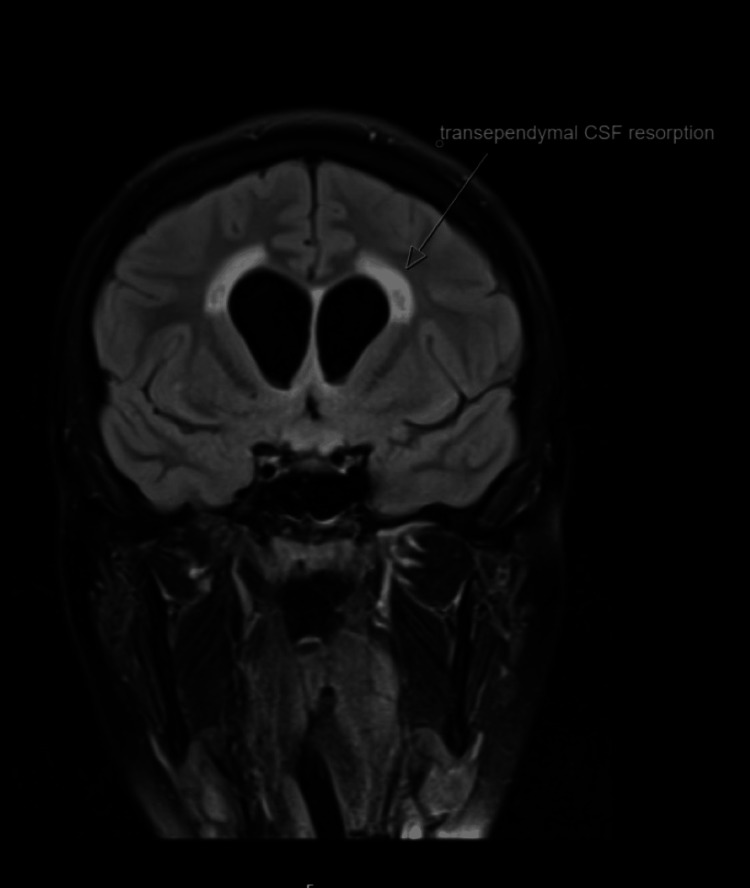
MRI of the head with contrast. White arrows: evidence of hydrocephalus.

The neurosurgical team obtained consent for an MRI under general anaesthesia. The MRI of the pituitary with contrast confirmed the findings of the CT scan, demonstrating both the pineal and the suprasellar masses along with the considerable mass effect and the acute obstructive hydrocephalus (Figures 5-7).

**Figure 8:**
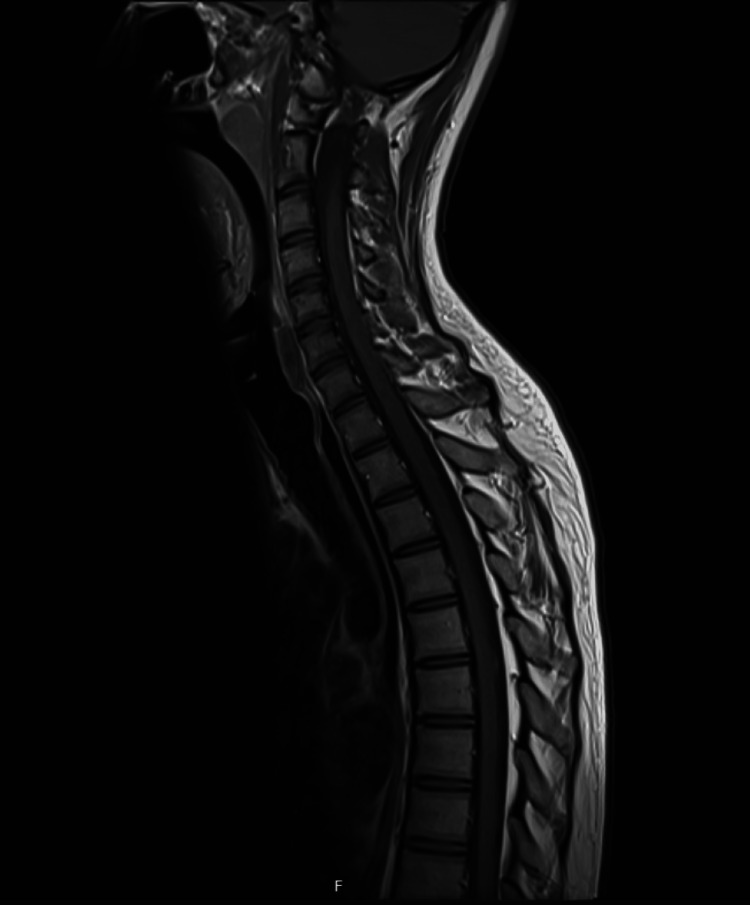
Normal MRI of the spine.

An MRI of the whole spine with contrast was also obtained, but was unremarkable (Figure 8).

**Figure 9:**
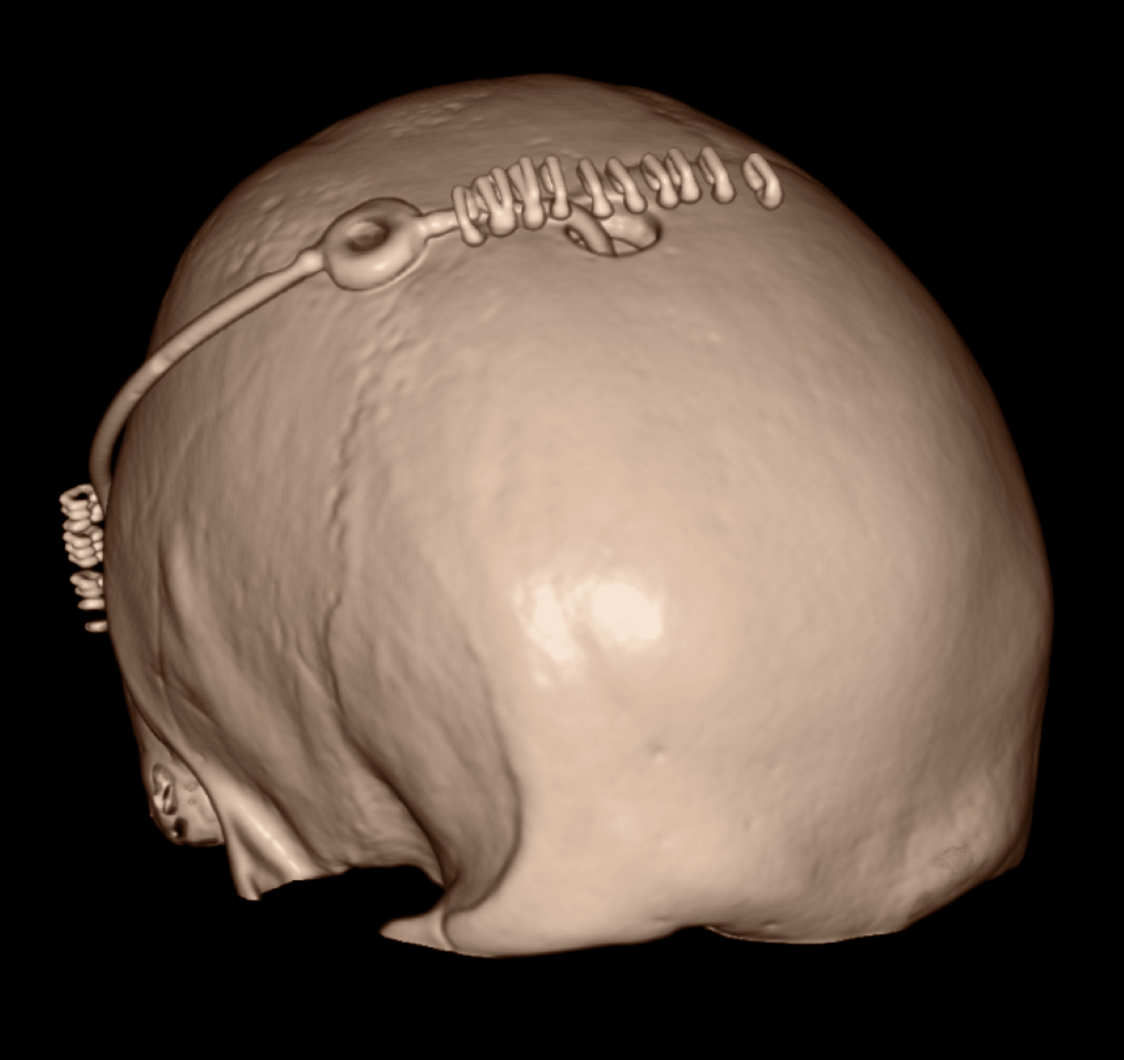
CT of the head following the insertion of the ventriculoperitoneal shunt.

**Figure 10:**
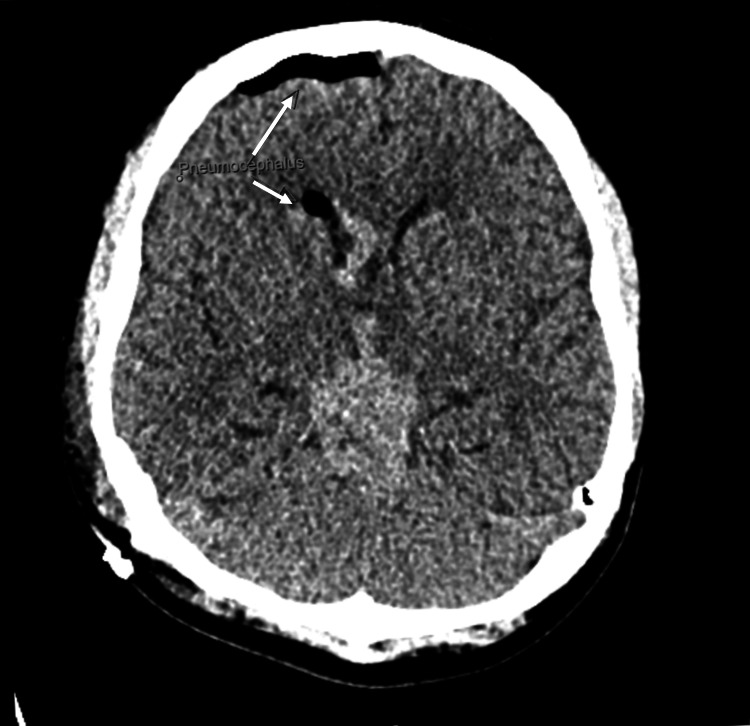
CT of the head following the insertion of the ventriculoperitoneal shunt. White arrows: pneumocephalus.

**Figure 11:**
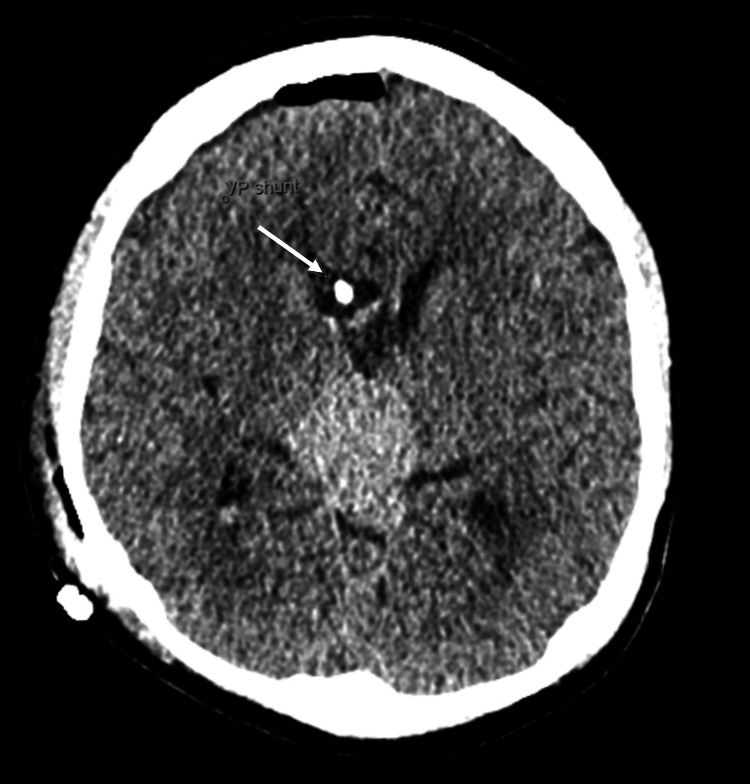
CT of the head following the insertion of the ventriculoperitoneal shunt. White arrow: ventriculoperitoneal shunt.

The neurosurgical team obtained consent for a right frontal endoscopic biopsy of the ventricular and suprasellar tumour, and a placement of a ventriculoperitoneal (VP) shunt, performed right after the MRI. A CT of the head was obtained after the surgery and showed the VP shunt in the frontal horn of the right lateral ventricle, with pneumocephalus, no active bleeding, and the previously known space-occupying lesion noted again (Figure 9-11).

**Figure 12:**
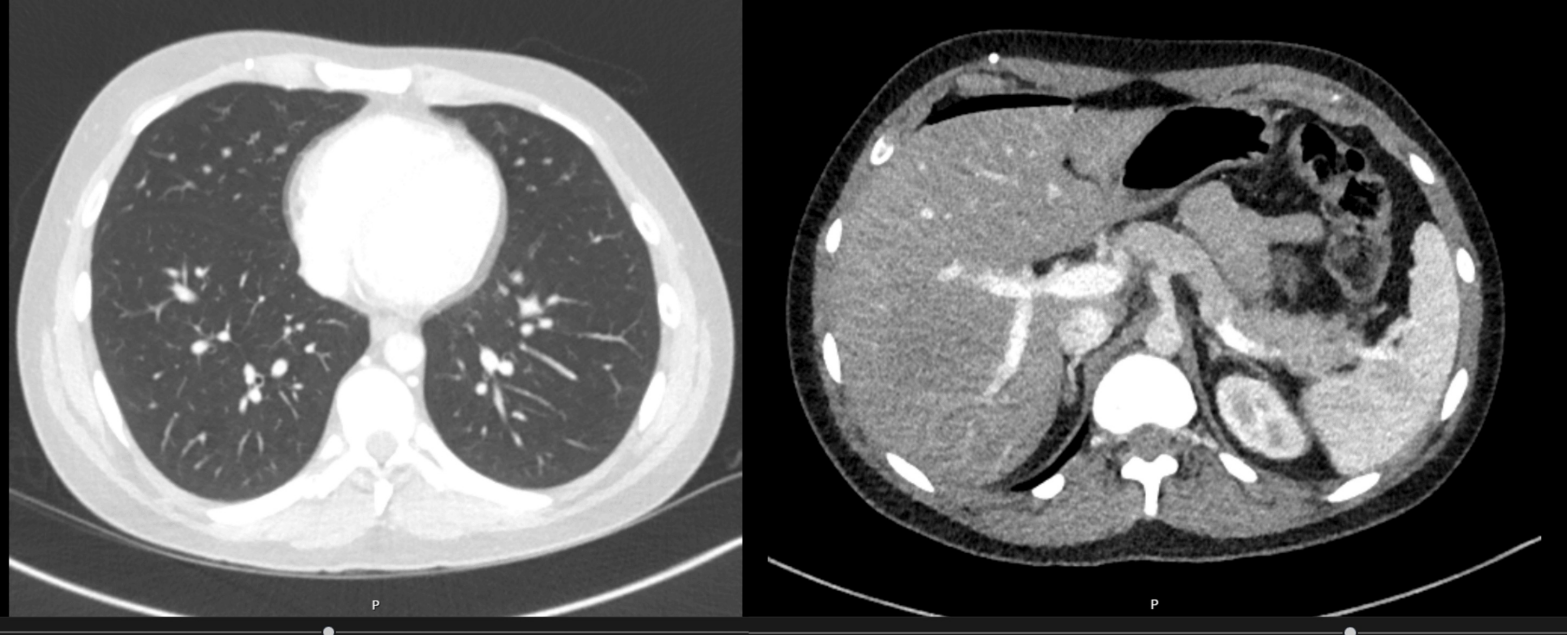
Normal CT of the thorax, abdomen, and pelvis.

**Figure 13:**
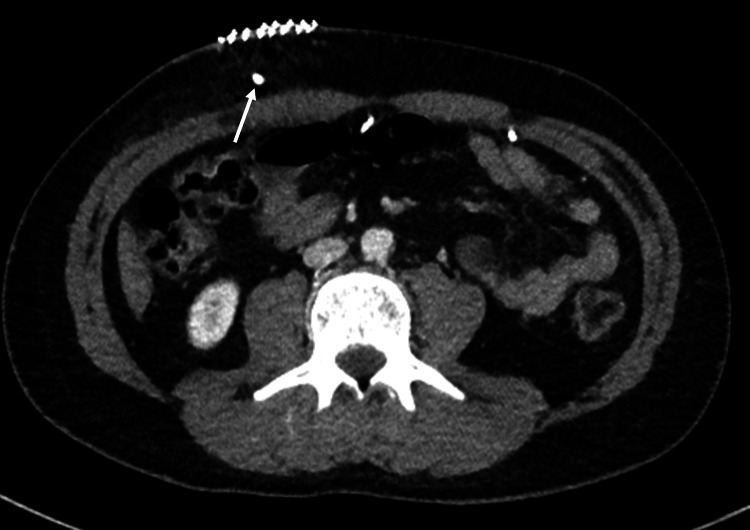
CT of the thorax, abdomen, and pelvis. White arrows: ventriculoperitoneal shunt.

The patient underwent a CT of the thorax, abdomen, and pelvis, which showed no metastasis and a well-positioned VP shunt (Figures 12, 13).

The findings of the CSF obtained during the operation are presented in Table 5.

**Table 5:** CSF results and serum biomarkers. CSF: cerebrospinal fluid; HCG: human chorionic gonadotropin; AFP: alpha-fetoprotein

Test	Result	Normal range
CSF AFP	<0.41 ng/mL	<1.5 ng/mL
CSF HCG	18 U/L	<1.0 U/L
Serum AFP	6.41 kU/L	<6 kU/L
Serum HCG	3.8 IU/L	2–3 IU/L

**Figure 14:**
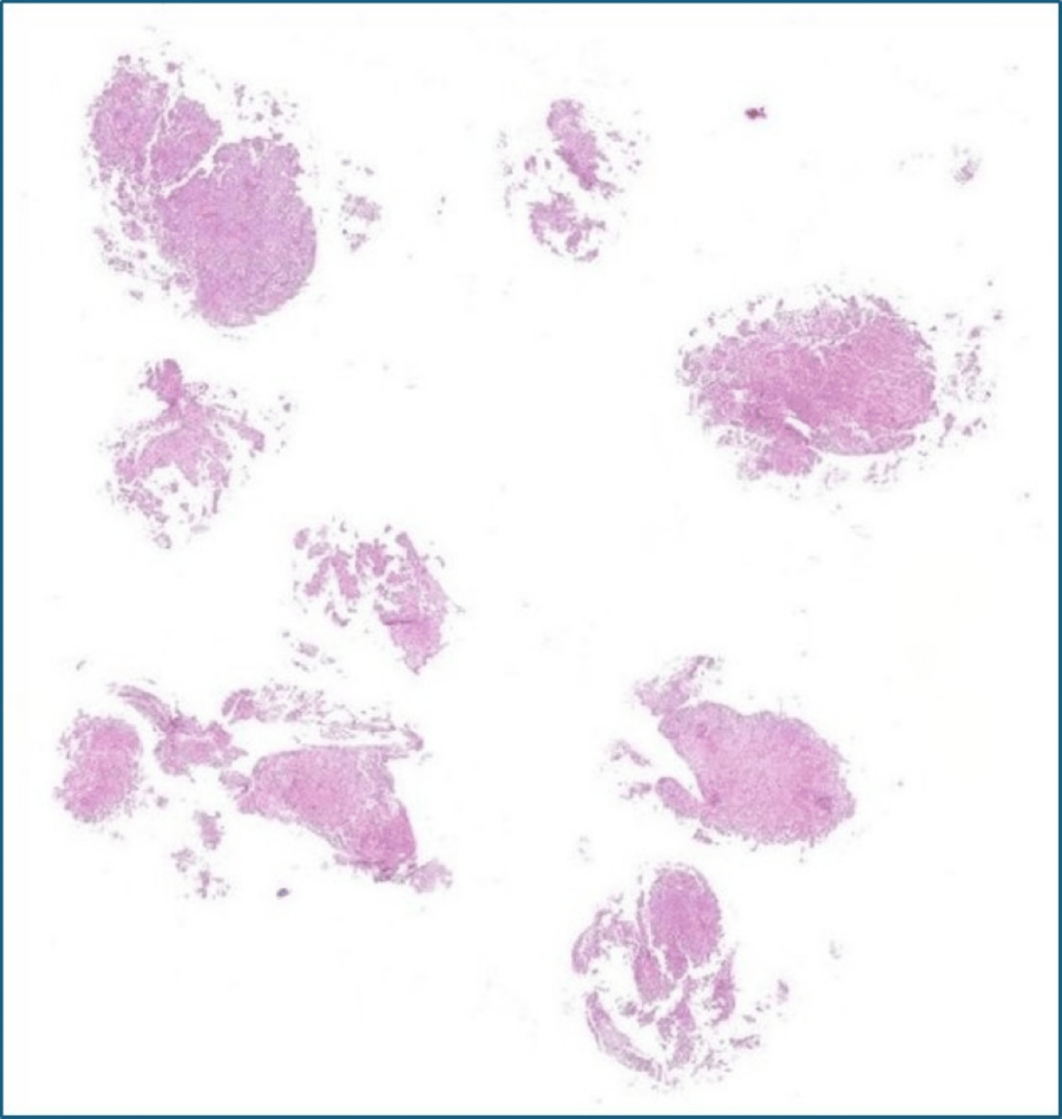
Tumor specimen from the third ventricle. Stain: hematoxylin and eosin; magnification: 40×. Fidings: The morphological and immunohistochemical appearances of the tumour in specimen A are entirely consistent with a germinoma. Occasional strips of ciliated cells are also noted.

The patient was referred to the neuro-oncology multidisciplinary team (MDT) to discuss the histology results shown in Figure 14.

The GCT medical oncology team started the patient on four cycles of neoadjuvant high-dose chemotherapy, alternating carboplatin, etoposide, and ifosfamide. He was discharged with plans for radiotherapy (possible proton therapy), with the exact details depending on chemotherapy response.

## Discussion

GCTs are neoplasms originating from primordial germ cells in the genital ridge, occurring at an early developmental stage during specification, migration, or colonisation of these cells [6]. GCT can arise in the gonads or elsewhere in the body (extragonadal), and they tend to occur in midline locations such as the mediastinum, parapineal and sacrococcygeal regions, and retroperitoneum [7].

GCTs have an incidence of 2.4 cases per million children per year [8] and represent fewer than 1% of all brain tumours in North America and Europe [9]. In the United Kingdom, fewer than 10 individuals a year develop intracranial GCTs [10], with incidence peaking between 10 and 19 years of age [11].

**Figure 15:**
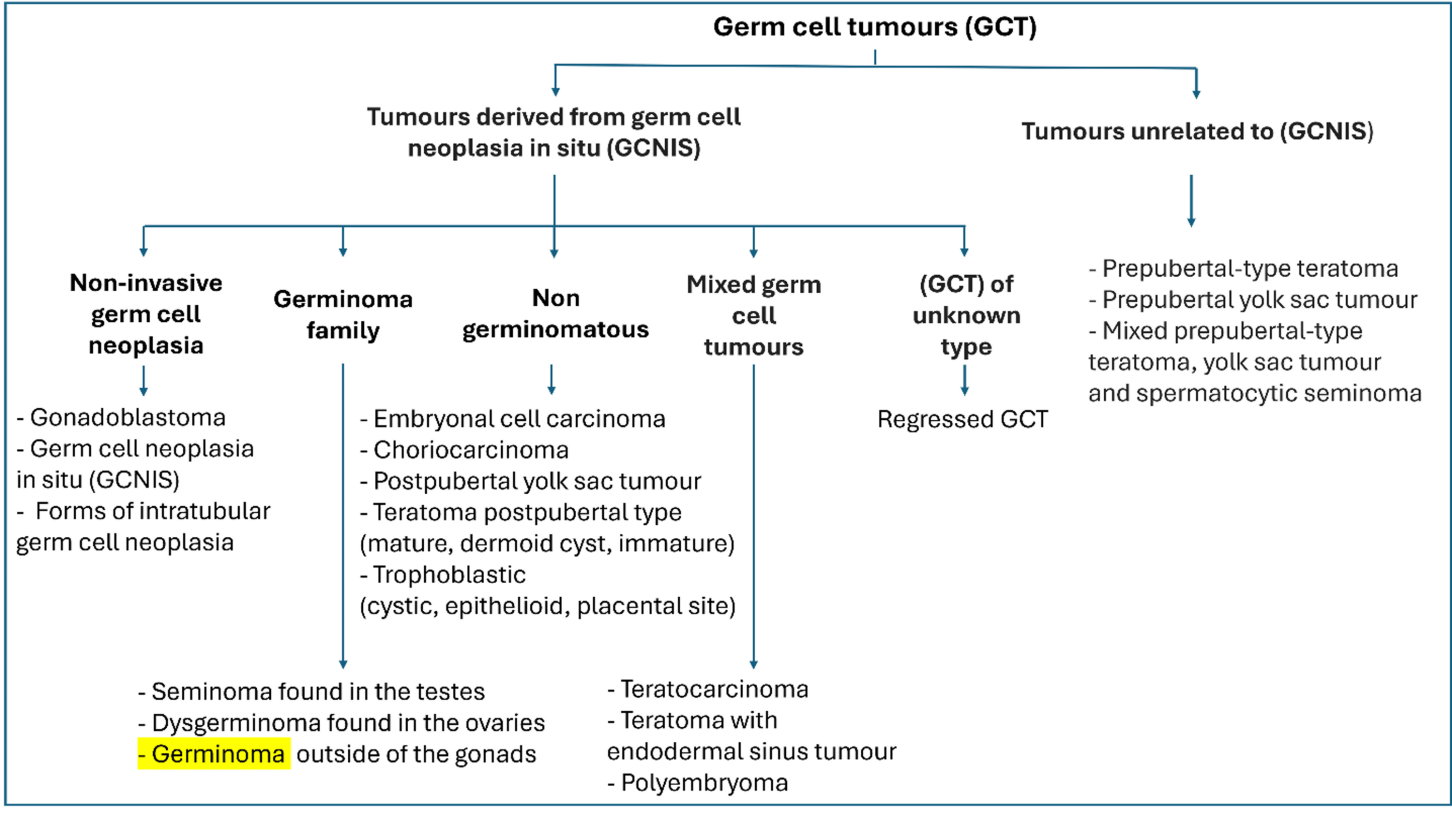
Classification of GCTs. Original image created by the authors. GCT: germ cell tumour

The World Health Organization introduced an update to the classification of GCTs in 2022, including terminology changes. The classification of GCTs is clarified in Figure 15, with germinoma highlighted in yellow [2,12-15].

The aetiology of GCTs as a whole and intracranial GCT remains a subject of exploration. Research is ongoing on the role of microenvironmental factors, chromosomal instability, testicular dysgenesis syndrome, epigenetic machinery, KIT/RAS alterations, and elevated KIT mRNA expression [6,16].

Testicular dysgenesis syndrome (TDS) addresses the strong correlation between testicular GCTs and other male reproductive disorders. It suggests that TDS is initiated in the developing foetus due to environmental factors, hormone-disrupting compounds, and genetic factors [17].

Studies have investigated the role of the KIT/RAS signalling pathway. Samples were screened for somatic mutation of *KIT*, *KRAS*, *NRAS*, *HRAS*, *BRAF*, *PDGFRA*, and *IDH1* by direct sequencing, with mutations detected in *KIT* and *RAS* in about 60% of pure germinomas (60.0%), but only 8.6% in non-germinomatous GCTs [16,18].

Genomic analysis has revealed a high frequency of regional gains and losses, including high-level gene amplification [19]. The patterns of methylation found in these genomic studies resemble those of germ cells, indicating a primordial origin. These cells originate in the yolk sac of the embryo during weeks three to four of gestation and are expected to be part of the gonads (the ovaries or testes). However, it is believed that GCTs can occur at specific extragonadal sites along the midline of the body, such as the pineal gland-hypothalamic region, mediastinum, retroperitoneum, and sacrum, due to the incorrect migration route of the primordial germ cells, which then become trapped in midline locations [20]. This belief is supported by the fact that CNS GCTs have similar DNA damage response activation patterns to those of gonadal (GCT) rather than those of CNS-originating tumours [21].

Germinomas

Germinomas are the most common intracranial GCTs and account for around 60-77% of cases. About 75% of germinomas in males occur in the pineal region, while 75% of germinomas appear in the sellar or suprasellar region in females [22]. Based on treatment options and prognosis, intracranial CGTs are divided into pure germinomas (approximately two-thirds of malignant cases) and non-germinomatous GCTs [23]. They can infiltrate the surrounding tissue or via CSF [24].

Manifestations depend on the tumour site and size, which can cause delayed diagnosis and variable symptoms, mimicking movement disorders or even psychiatric disease [25]. Patients can present with generalised symptoms such as lethargy, loss of appetite or weight, altered sleep patterns, behavioural problems, mood problems, and poor scholastic performance.

Basal ganglia and thalamus germinomas usually present late with slow, progressive, vague symptoms, especially limb weakness, which can progress to hemiplegia, headaches, and visual disturbances [26]. The vague nature of the symptoms, coupled with the rarity of the condition, can lead to delayed diagnosis.

Pineal intracranial GCTs usually cause cognitive decline and a wide range of neurological symptoms, as they tend to increase intracranial pressure. This is due to the anatomical site of pineal intracranial GCT, leading to compression of the cerebral aqueduct, causing obstructive hydrocephalus. Patients then complain of headaches, vomiting, visual disturbance, and seizures [27,28]. Patients can develop Parinaud’s syndrome, which is characterised by upward gaze paralysis, light-near dissociation, and convergence-retraction nystagmus [22].

On the other hand, sellar and suprasellar intracranial GCTs usually cause hypothalamo-hypophyseal insufficiency with clinical manifestations relating to the corresponding endocrine abnormalities. Patients may first present with one of those endocrinopathies rather than neurological manifestations.

Studies show endocrine deficits were present in 63.5% of the cases at diagnosis [29]. Other researchers even found that pituitary deficits were present in all patients studied [30]. These endocrine deficits can persist or even worsen after tumour remission, which is thought to be secondary to radiation [31]. Examples of endocrinopathies that can occur with intracranial GCT are diabetes insipidus, growth hormone deficiency, central hypothyroidism, stunted growth, central adrenal insufficiency, hypogonadotropic hypogonadism, delayed sexual development, galactorrhoea, and menstrual irregularity. Patients typically have a collection of symptoms [32-34]. Growth delay can be the earliest sign of intracranial GCTs. Early diagnosis of hypothyroidism and growth hormone deficiency, as the two main endocrinological causes of growth delay, can lead to a good prognosis and early diagnosis of intracranial GCTs [35,36].

Symptoms of associated endocrinopathies

The most common symptoms of central hypothyroidism are fatigue and headaches in patients with adult-onset central hypothyroidism and growth retardation in those who were diagnosed as children. Other possible symptoms are similar to those of primary hypothyroidism, such as drowsiness, adynamia, skin dryness, cold intolerance, constipation, generalised lethargy, and weight gain [37,38].

In central adrenal insufficiency (CAI), the classic clinical signs of Addison’s, such as skin pigmentation, may be absent, as there is no ACTH excess. Symptoms vary depending on other associated pituitary deficiencies, severity, and time of onset. Patients can be asymptomatic, have non-specific symptoms, as fatigue, weakness, poor appetite, dizziness, or be extremely unwell with vomiting, hypoglycaemia, and hypotension during an adrenal crisis [39,40]. It is worth noting that symptoms of central diabetes insipidus, such as polyuria and polydipsia, can be vague and only be unmasked after starting glucocorticoid replacement therapy [41].

Central hypogonadotropic hypogonadism can cause delayed or even absent sexual development and infertility. Newborn boys present with a triad of micropenis, cryptorchidism, and microorchidism, while newborn girls have no obvious abnormalities. In children, there is a lack of growth and sexual development at the standard age for puberty. Girls have an absence of breast development and menstruation, while boys have no sex characteristics, including facial hair, deepening of the voice, and enlargement of the gonads. In adults, there is secondary amenorrhea, reduced libido, infertility, and osteoporosis in women and symptoms of reduced libido, erectile dysfunction, fatigue, and infertility in men [42,43].

In adult growth hormone deficiency, patients report anxiety, depression, obesity, loss of muscle bulk, reduced exercise tolerance, dyslipidaemia, osteoporosis, cardiac complications and overall lack of general well-being. In children, patients show growth retardation, short stature, hypoglycaemia, and maturation delays [44,45].

Diagnosing intracranial GCTs depends heavily on imaging and serum and CSF biomarkers, yet pathology from a surgical biopsy or a resection specimen remains the gold standard [46]. In terms of imaging, on CT, intracranial GCTs enhance brightly due to their high cellularity, resulting in hyperdensity. On MRI, intracranial GCTs can be seen as a soft tissue mass, typically ovoid or lobulated in contour, with areas of cyst formation, haemorrhage, or invading adjacent brain with oedema [47].

Regarding biomarkers, GCTs may secrete specific circulating tumour markers, including AFP and HCG. Increased serum or CSF markers can precede the radiological finding by several months, resulting in a high predictive/diagnostic power [48]. These biomarkers are beneficial in differentiating pure germinomas from non-germinomatous GCTs, as a pure germinoma usually shows no rise in AFP or HCG [49].

Regarding pathology, the findings depend on the type of intracranial GCT. On macroscopical examination, pure germinomas are pale grey solid nodules, with less haemorrhage, necrosis, and cystic change. Microscopically, they show sheets or trabeculae of large, undifferentiated cells with a round central nucleus and clear, abundant cytoplasm. Lymphocytic infiltration can occur, but necrosis is rare. Non-germinomatous GCTs, however, show different findings depending on their type; for example, yolk sac tumours show primitive looking epithelium with myxoid matrix, and embryonal carcinomas can show nests of large cells with some necrosis [50,51].

Diagnosis of endocrinopathies secondary to intracranial GCTs can precede the diagnosis of intracranial GCTs if patients present with endocrinopathy-related symptoms first. The diagnosis of central hypothyroidism can be missed if a TSH is the only screening test used, as the TSH produced can be biologically inactive and affect the levels of bioactive TSH; thus, it is recommended to measure free T4. Imaging is not routinely done; however, antithyroid antibodies such as thyroid peroxidase antibodies should also be assessed to evaluate for autoimmune thyroid diseases, especially if this is the first presentation without a diagnosis. It is extremely important to assess the pituitary adrenal axis before starting levothyroxine to avoid precipitation of adrenal crisis in patients with adrenal insufficiency [38,52].

The use of basal cortisol to diagnose CAI requires care, as there are many pitfalls in laboratory measurements. However, generally, an 8 am cortisol level of >300 nmol/L indicates that the hypothalamic-pituitary-adrenal axis is functioning normally. If cortisol levels are lower, an additional dynamic pituitary stimulation test is necessary. The test is done using recombinant ACTH (Synacthen or Cortrosyn) as a stimulus and measuring cortisol levels at 30 minutes, expecting a level of >367 nmol/L as a normal response. False positives can occur if the test is performed in the acute phase (less than six months of disease). Once the laboratory diagnosis of CAI is confirmed, an imaging evaluation of the pituitary is necessary [39,53].

Regarding central diabetes insipidus, the first step in the diagnosis is to confirm the presence of hypotonic polyuria. A urine osmolality >700 mOsm/kg or a urine volume <2.5 L/day in an adult rules out central diabetes insipidus. Other causes also need to be ruled out, such as diabetes mellitus, renal impairment, hyperglycaemia, and hypercalcemia. Plasma sodium is almost always normal at diagnosis in central diabetes insipidus. The next step would be to perform a two-step water deprivation test with an eight-hour period of water deprivation followed by administration of parenteral desmopressin and measuring plasma and urine osmolarities. Urine osmolality of less than 300 mOsmol/kg after fluid deprivation and greater than 800 mOsmol/kg after desmopressin suggests cranial diabetes insipidus [54-56].

Diagnosing central hypogonadotropic hypogonadism starts with measuring hormone levels, including morning total testosterone, FSH, LH, TSH, prolactin, and oestradiol. The next step would be to send the rest of the pituitary panel, consider genetic testing, and an MRI of the pituitary. Renal ultrasound can be requested in case of suspicion of Kallmann syndrome [43,44].

The diagnosis of growth hormone deficiency is multifactorial. Measurement of random serum growth hormone concentrations is of no clinical value, as growth hormone secretion is pulsatile. While insulin-like growth factor-1 is a good alternative, multiple conditions affect its levels, such as diabetes and renal disease, with its concentration varying with age. Therefore, testing should proceed with a growth hormone stimulation test, which uses a defined cut-off concentration for peak growth hormone to distinguish growth hormone deficiency from non-growth hormone deficiency subjects. Examples of these tests include the insulin tolerance test, arginine, glucagon, clonidine, pyridostigmine, levodopa, growth hormone-releasing hormone, and growth hormone-releasing hormone combined. It is recommended not to use growth hormone stimulation tests as a sole diagnostic tool, but to use them along with physical findings, screening tests, and insulin-like growth factor-1 and insulin-like growth factor-BP3 levels. A pituitary MRI is required in all confirmed patients [57,58]. It is recommended to treat adults with growth hormone deficiency only if they fulfil all three of the following criteria: peak growth hormone response of less than 9 mU/L (3 ng/mL) during an insulin tolerance test or an equivalent test, Quality of Life Assessment of Growth Hormone Deficiency in Adults questionnaire score showing an impaired quality of life, and on treatment for any other pituitary deficiency [59].

Treatment

Treatment options depend on the type of CNS GCT. Complete surgical resection does not play a major role in the management of these highly vascular tumours. Germinomas are very responsive to chemotherapy and radiation. However, chemotherapy alone is insufficient to allow for acceptable cure rates, about 50% only. On the other hand, high doses of radiation alone can provide a curative option for the majority of patients, around 90%. It used to be the gold standard for treating germinomas in the form of craniospinal irradiation. However, the late side effects of radiation, especially in growing individuals, have shifted management protocols towards combining chemotherapy with lower volumes and doses of radiation therapy. This reduces the adverse effects of radiation and improves the quality of life of patients who develop neurologic, neurocognitive, and/or endocrine deficiencies [24,60,61].

Prognosis

Even though there are differences in the management of intracranial GCTs globally, the current survival rates are 80-100% for germinomas and 60-80% for non-germinomatous with multimodality treatment. Germinomas have a good prognosis, and the five-year overall survival rate is more than 90% [24,60,61].

## Conclusions

This case report highlights the significance of including uncommon central causes in the differential diagnosis of new-onset endocrinopathies, particularly in young adults, even if they achieved normal growth milestones earlier in life. Diagnosing this condition in adults is challenging due to its rarity and diverse presentations. For this reason, it is crucial to consult a specialist promptly if these causes are suspected, so that proper investigations can be performed and early treatment can begin. The involvement of a multidisciplinary team was crucial to achieving an accurate diagnosis in this case, and effective management of this multisystem disease relied on their collaborative efforts. The treatment described above is a lengthy process; therefore, ongoing follow-up is highly important.

## References

[1] Echevarría ME, Fangusaro J, Goldman S (2008). Pediatric central nervous system germ cell tumors: a review. Oncologist.

[2] Moch H, Amin MB, Berney DM (2022). The 2022 World Health Organization Classification of Tumours of the Urinary System and Male Genital Organs-Part A: renal, penile, and testicular tumours. Eur Urol.

[3] Takami H, Graffeo CS, Perry A (2022). Roles of tumor markers in central nervous system germ cell tumors revisited with histopathology-proven cases in a large international cohort. Cancers (Basel).

[4] Oosterhuis JW, Looijenga LH (2005). Testicular germ-cell tumours in a broader perspective. Nat Rev Cancer.

[5] Murray MJ, Coleman N (2012). Testicular cancer: a new generation of biomarkers for malignant germ cell tumours. Nat Rev Urol.

[6] Müller MR, Skowron MA, Albers P, Nettersheim D (2021). Molecular and epigenetic pathogenesis of germ cell tumors. Asian J Urol.

[7] Talerman A (1985). Germ cell tumours. Ann Pathol.

[8] Rodriguez-Galindo C, Pappo AS., Kufe DW (2003). Holland-Frei Cancer Medicine. 6th Edition. Holland-Frei Cancer Medicine. 6th.

[9] (2025). Brain Tumour Research. Germ cell tumour (GCT). https://braintumourresearch.org/pages/types-of-brain-tumours-germ-cell-tumour-gct.

[10] (2025). The Royal Marsden. Intracranial germ cell tumour. https://www.royalmarsden.nhs.uk/your-care/cancer-types/paediatric-cancers/germ-cell-tumours/intracranial-germ-cell-tumour.

[11] Frappaz D, Dhall G, Murray MJ (2022). EANO, SNO and Euracan consensus review on the current management and future development of intracranial germ cell tumors in adolescents and young adults. Neuro Oncol.

[12] Berney DM, Cree I, Rao V (2022). An introduction to the WHO 5th edition 2022 classification of testicular tumours. Histopathology.

[13] Al-Obaidy KI, Idrees MT (2021). Testicular tumors: a contemporary update on morphologic, immunohistochemical and molecular features. Adv Anat Pathol.

[14] Bode PK, Blasco-Santana L, Colmenero I, Reyes-Múgica M (2025). Germ cell tumors in children. Virchows Arch.

[15] (2025). Pathology Outlines. Testis & paratestis general WHO classification. https://www.pathologyoutlines.com/topic/testistestclassif.html.

[16] Fukushima S, Otsuka A, Suzuki T (2014). Mutually exclusive mutations of KIT and RAS are associated with KIT mRNA expression and chromosomal instability in primary intracranial pure germinomas. Acta Neuropathol.

[17] Sonne SB, Kristensen DM, Novotny GW (2008). Testicular dysgenesis syndrome and the origin of carcinoma in situ testis. Int J Androl.

[18] McIntyre A, Summersgill B, Grygalewicz B (2005). Amplification and overexpression of the KIT gene is associated with progression in the seminoma subtype of testicular germ cell tumors of adolescents and adults. Cancer Res.

[19] Schulte SL, Waha A, Steiger B (2016). CNS germinomas are characterized by global demethylation, chromosomal instability and mutational activation of the Kit-, Ras/Raf/Erk- and Akt-pathways. Oncotarget.

[20] Elzinga-Tinke JE, Dohle GR, Looijenga LH (2015). Etiology and early pathogenesis of malignant testicular germ cell tumors: towards possibilities for preinvasive diagnosis. Asian J Androl.

[21] Fukushima S, Yamashita S, Kobayashi H (2017). Genome-wide methylation profiles in primary intracranial germ cell tumors indicate a primordial germ cell origin for germinomas. Acta Neuropathol.

[22] Kremenevski N, Buchfelder M, Hore N (2023). Intracranial germinomas: diagnosis, pathogenesis, clinical presentation, and management. Curr Oncol Rep.

[23] (2025). European Reference Network. Childhood intracranial germ cell tumours. Cell Tumours: Dr Manuel Diezi, Professor Barry Pizer, and Professor Matthew Murray. Published.

[24] Osorio DS, Allen JC (2015). Management of CNS germinoma. CNS Oncol.

[25] Crawford JR, Santi MR, Vezina G (2007). CNS germ cell tumor (CNSGCT) of childhood: presentation and delayed diagnosis. Neurology.

[26] Tuan HX, Huyen NT, Son ND, Trung NV, Anh NH, Hung ND, Duc NM (2024). Germinoma of basal ganglia. Radiol Case Rep.

[27] Jorsal T, Rørth M (2012). Intracranial germ cell tumours. A review with special reference to endocrine manifestations. Acta Oncol.

[28] Wataya T, Ishizaki R, Kitagawa M, Tashiro Y (2015). Germinoma in the bilateral basal ganglia presented with cognitive deterioration. Childs Nerv Syst.

[29] Poon SW, Tung JY, Pang GS (2025). Evaluating diagnostic delays and late endocrine sequelae in children with intracranial germ cell tumors: a territory-wide population-based study. Pediatr Blood Cancer.

[30] Sklar CA, Grumbach MM, Kaplan SL, Conte FA (1981). Hormonal and metabolic abnormalities associated with central nervous system germinoma in children and adolescents and the effect of therapy: report of 10 patients. J Clin Endocrinol Metab.

[31] Sa ki N, Tamaki K, Murai H (2000). Long-term outcome of endocrine function in patients with neurohypophyseal germinomas. Endocr J.

[32] Buchfelder M, Fahlbusch R, Walther M, Mann K (1989). Endocrine disturbances in suprasellar germinomas. Acta Endocrinol (Copenh).

[33] Partenope C, Pozzobon G, Weber G, Arya VB, Carceller F, Albanese A (2022). Endocrine manifestations of paediatric intracranial germ cell tumours: from diagnosis to long-term follow-up. Endocrine.

[34] Chang HY, Lo FS (2020). MON-085 The clinical and endocrinologic manifestations of germinoma in Taiwanese pediatric population: one medical center experience. J Endocr Soc.

[35] Millichap JG (2006). Suprasellar germinoma and growth retardation. Pediatr Neurol Briefs.

[36] Gottschling S, Graf N, Meyer S, Reinhard H, Krenn T, Rohrer T (2006). Intracranial germinoma: a rare but important differential diagnosis in children with growth retardation. Acta Paediatr.

[37] Gupta V, Lee M (2011). Central hypothyroidism. Indian J Endocrinol Metab.

[38] Gudmundsdottir A, Schlechte JA (2002). Central hypothyroidism. Endocrinologist.

[39] Bitencourt MR, Batista RL, Biscotto I, Carvalho LR (2022). Central adrenal insufficiency: who, when, and how? From the evidence to the controversies - an exploratory review. Arch Endocrinol Metab.

[40] Ceccato F, Scaroni C (2019). Central adrenal insufficiency: open issues regarding diagnosis and glucocorticoid treatment. Clin Chem Lab Med.

[41] Chin HX, Quek TP, Leow MK (2017). Central diabetes insipidus unmasked by corticosteroid therapy for cerebral metastases: beware the case with pituitary involvement and hypopituitarism. J R Coll Physicians Edinb.

[42] Marino M, Moriondo V, Vighi E, Pignatti E, Simoni M (2014). Central hypogonadotropic hypogonadism: genetic complexity of a complex disease. Int J Endocrinol.

[43] (2025). University of Florida Health. Hypogonadotropic hypogonadism. https://ufhealth.org/conditions-and-treatments/hypogonadotropic-hypogonadism.

[44] Johannsson G, Ragnarsson O (2021). Growth hormone deficiency in adults with hypopituitarism-what are the risks and can they be eliminated by therapy?. J Intern Med.

[45] (2025). Growth hormone deficiency. https://rarediseases.org/rare-diseases/growth-hormone-deficiency/.

[46] Huang L, Gong J, Feng D (2025). A comprehensive dataset of germinoma on MRI/CT with clinical and radiomic data. Sci Data.

[47] Gaillard F, Agazzi G, Dieckmeyer M (2025). Radiopaedia. Central nervous system germinoma. https://radiopaedia.org/articles/central-nervous-system-germinoma?lang=us.

[48] D'Alessandro A, Ciavardelli D, Pastore A (2021). Cerebrospinal fluid levels of AFP and hCG: validation of the analytical method and application in the diagnosis of central nervous system germ cell tumors. Diagnostics (Basel).

[49] Gaillard F, Ghimire P, Kang O (2025). Radiopaedia. Intracranial germ cell tumours. https://doi.org/10.53347/rID-5956.

[50] Diezi M, Pizer B, Murray MJ (2024). Overview of current European practice for the management of patients with intracranial germ cell tumours. EJC Paediatr Oncol.

[51] Gao Y, Jiang J, Liu Q (2014). Clinicopathological and immunohistochemical features of primary central nervous system germ cell tumors: a 24-years experience. Int J Clin Exp Pathol.

[52] Patil N, Rehman A, Anastasopoulou C (2025). Hypothyroidism. https://www.ncbi.nlm.nih.gov/books/NBK519536/.

[53] (2025). Imperial Centre for Endocrinology. Endocrinology handbook. https://www.impendo.co.uk/endocrine-bible.

[54] Garrahy A, Moran C, Thompson CJ (2019). Diagnosis and management of central diabetes insipidus in adults. Clin Endocrinol (Oxf).

[55] Mutter CM, Smith T, Menze O, Zakharia M, Nguyen H (2021). Diabetes insipidus: pathogenesis, diagnosis, and clinical management. Cureus.

[56] (2025). Water deprivation and desmopressin test. https://gpnotebook.com/en-GB/pages/diabetes-and-endocrinology/water-deprivation-and-desmopressin-test.

[57] Yau M, Rapaport R (2022). Growth hormone stimulation testing: to test or not to test? That is one of the questions. Front Endocrinol (Lausanne).

[58] Murray PG, Dattani MT, Clayton PE (2016). Controversies in the diagnosis and management of growth hormone deficiency in childhood and adolescence. Arch Dis Child.

[59] (2025). Human growth hormone (somatropin) in adults with growth hormone deficiency. https://www.nice.org.uk/guidance/ta64/chapter/1-Recommendations.

[60] Zhou Z, Liu J, Yue Q (2025). Optimal treatment approach for intracranial germinoma: a systematic review and meta-analysis. BMC Cancer.

[61] Bowzyk Al-Naeeb A, Murray M, Horan G, Harris F, Kortmann RD, Nicholson J, Ajithkumar T (2018). Current management of intracranial germ cell tumours. Clin Oncol (R Coll Radiol).

